# 48 HOUR HOSPICE HOME IMMERSION RESEARCH KEY MEDICAL STUDENT LEARNING OUTCOMES: UNE COM 2019-20 STUDENT COHORT

**DOI:** 10.1093/geroni/igae098.3772

**Published:** 2024-12-31

**Authors:** Amanda Okpoebo, Jennifer Berkowitz, Marilyn Gugliucci

**Affiliations:** University of New England College of Osteopathic Medicine, Biddeford, Maine, United States; University of New England College of Osteopathic Medicine, Biddeford, Maine, United States; University of New England College of Osteopathic Medicine, Biddeford, Maine, United States

## Abstract

Traditional palliative and end-of-life care education in US medical schools is often inadequate in preparing students to care for patients. To address this, the University of New England College of Osteopathic Medicine (UNE COM) introduced the Learning by Living 48-Hour Hospice Home Immersion (HHI) Project. This innovative program immerses students into an 18-bed acute care (ICU) hospice home for 48 hours, where they provide care to patients, support families, and engage in postmortem care while working with an interprofessional staff team and independently. Utilizing qualitative ethnographic/autobiographic research designs, two key research questions included: (1) What significant lessons were learned from the 48-hour home hospice immersion?; and (2) What skills will they apply in their future practice? Students documented their experiences in journals. Eleven second-year medical student journals from the 2019-20 cohort were selected for analysis; 300+ pages of data. During each of three stages: pre-fieldwork, fieldwork, and post-fieldwork, qualitative analysis included content review, creating a codebook, and conducting a final analysis with NVIVO 14 software. Three key themes emerged for question one: (a) Holistic comfort care/medicine, (b) Subjectivity of suffering, and (c) Religion/spirituality. For question two, students reported: (a) Increased confidence in caring for dying patients and their families, (b) Improved ability to discuss death, and (c) Enhanced understanding of the physician’s role in curing versus caring. Each student’s experience at Gosnell Hospice Home was unique, yet all gained critical insights that will shape their development into compassionate and skilled physicians in palliative and end-of-life care.

